# Supplementary material for: Anisotropic moiré optical transitions in twisted monolayer/bilayer phosphorene heterostructures
Source: Nat Commun. 2021 Jun 24;12:3947. doi: 10.1038/s41467-021-24272-9 (PMC8225781; doi:10.1038/s41467-021-24272-9)
Supplement: Supplementary file 1 — Supplementary Information [file 41467_2021_24272_MOESM1_ESM.pdf]

# Supplementary Information for

**Anisotropic Moiré Optical Transitions in Twisted Monolayer/bilayer Phosphorene Heterostructures**

Shilong Zhao[1,2], Erqing Wang[1], Ebru Alime Üzer[3], Shuaifei Guo[4], Ruishi Qi[2,5], Junyang Tan[1], Kenji Watanabe[6], Takashi Taniguchi[6], Tom Nilges[3], Peng Gao[5,7], Yuanbo Zhang[4], Hui-Ming Cheng[1], Bilu Liu[1*], Xiaolong Zou[1*] & Feng Wang[2,8,9]*

These authors contributed equally: Shilong Zhao, Erqing Wang

* Correspondence to: bilu.liu@sz.tsinghua.edu.cn, xlzou@sz.tsinghua.edu.cn, or fengwang76@berkeley.edu.

Supplementary Note 1. The moiré superlattice in twisted monolayer/bilayer phosphorene heterostructures

Supplementary Note 2. Atom configurations for special stacking configurations

Supplementary Note 3. Identification of twisted monolayer/bilayer phosphorene heterostructures

Supplementary Note 4. Angle determination for twisted monolayer/bilayer phosphorene heterostructures

Supplementary Note 5. Relationship between the twist angle and lowest energy transitions

Supplementary Note 6. Determine the primary optical axis for the 20° twisted monolayer/bilayer phosphorene heterostructure

Supplementary Note 7. Calculated band structures and the imaginary part of dielectric function for few-layer phosphorenes

Supplementary Note 8. Partial charge distributions of few-layer phosphorenes

Supplementary Note 9. Calculated properties of twisted monolayer/bilayer phosphorene heterostructures

Supplementary Note 1. The moiré superlattice in twisted monolayer/bilayer phosphorene heterostructures

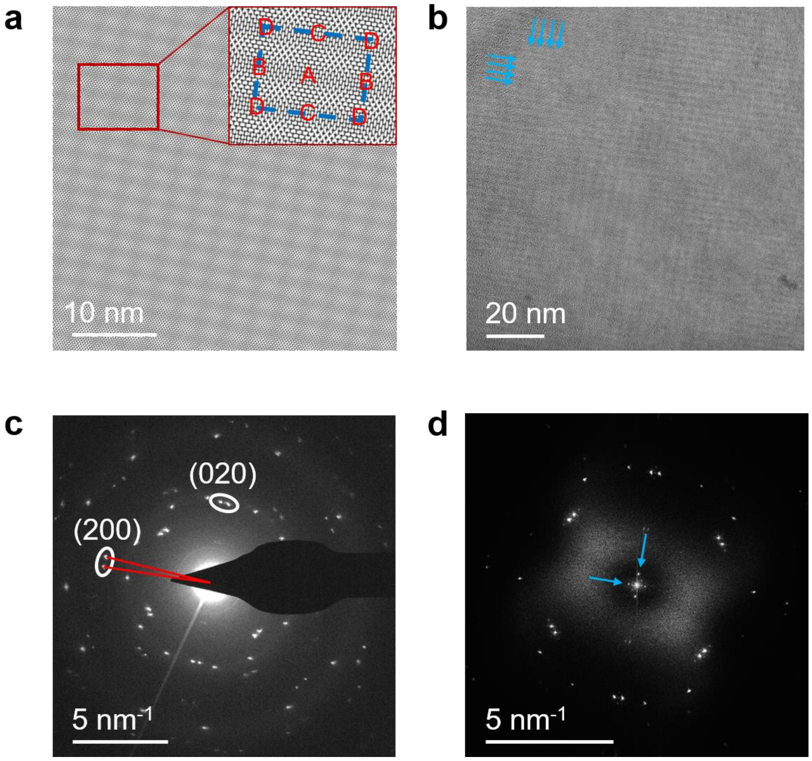


Supplementary Fig. 1. **HRTEM measurements of a twisted monolayer/bilayer phosphorene heterostructure with an angle of ~4.5°. a**, The top view of a structural model for a 4.5° twisted monolayer/bilayer phosphorene heterostructure, which shows clearly two orthogonal periods. The blue dashed lines in the inset zoom-in image display the rectangular shape moiré superlattice formed by four different stacking configurations, namely near ABA, ABB, ABC, and ABD stacking, labeled as A, B, C, and D, respectively. It indicates that moiré supercell periodicities are twice as that for apparent modulation period. **b**, The bright-field HRTEM image shows moiré superlattices along two directions, as indicated by the blue arrows. **c**, The selected area electron diffraction (SAED) patterns. Two sets of phosphorene diffraction patterns rotated by an angle of ~4.5°, as indicated by the red lines, can be easily identified. **d**, The Fourier transform image of **b**.

The blue arrows label two spots associated with periodicities of the apparent modulation period in **b**, from which the moiré periodicities in two directions (blue arrows in **b**) can be determined as ~4.4 nm and ~6.0 nm, respectively.

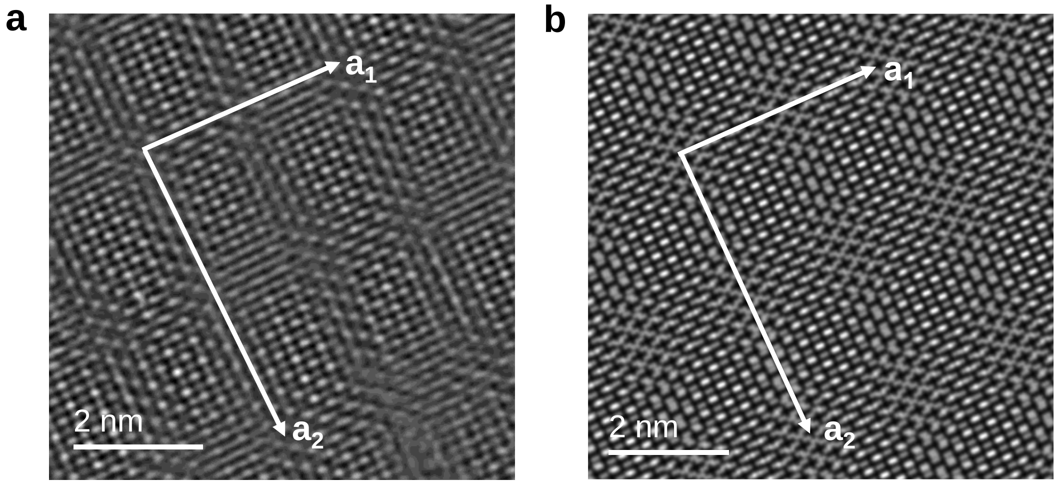


Supplementary Fig. 2. **HAADF-STEM images of a twisted monolayer/bilayer phosphorene heterostructure with an angle of ~5.7°. a**, The experimental HAADF-STEM image of a twisted monolayer/bilayer phosphorene. It shows clearly rectangular shape moiré superlattices where two superlattice vectors are labeled as $\mathbf{a}_1$ and $\mathbf{a}_2$, respectively. **b**, Top view of a simulated moiré superlattice for the same structure in **a**. Each phosphorus atom is modeled as a 2D Gaussian peak. This can be directly compared with the HAADF-STEM image, because this model is essentially a linear projection of phosphorus atoms along the beam direction. Two vectors of moiré superlattices, namely $\mathbf{a}_1$ and $\mathbf{a}_2$ (white arrows), have a length of ~3.2 nm and ~4.4 nm, respectively. Since there is a large intensity contrast between phosphorus and carbon atoms ($I_P/I_C$ ~ 6) in HAADF-STEM images, the observed moiré pattern is formed by the twisted monolayer/bilayer phosphorene rather than graphene/phosphorene interface.

Supplementary Note 2. Atom configurations for special stacking configurations

The intrinsic stacking order of few-layer phosphorene is ABA stacking, as illustrated in Supplementary Fig. 3a. The relative translation between the top monolayer and bottom bilayer phosphorene gives rise to different stacking orders, see Supplementary Fig. 3 (b–f). The 20° rotation between monolayer and bilayer phosphorene layers in large-angle twisted phosphorene heterostructure leads to four special stacking configurations, namely A, B, C, and D configurations (Fig. 1c in the main text). The atom configurations in A, B, C, and D are close to the perfectly aligned ABA (Supplementary Fig. 3c), ABB (Supplementary Fig. 3d), ABC (Supplementary Fig. 3e), and ABD stacking (Supplementary Fig. 3f), respectively, and are referred as near-ABA (Supplementary Fig. 4a), near-ABB (Supplementary Fig. 4b), near-ABC (Supplementary Fig. 4c), and near-ABD stacking configurations (Supplementary Fig. 4d), respectively.

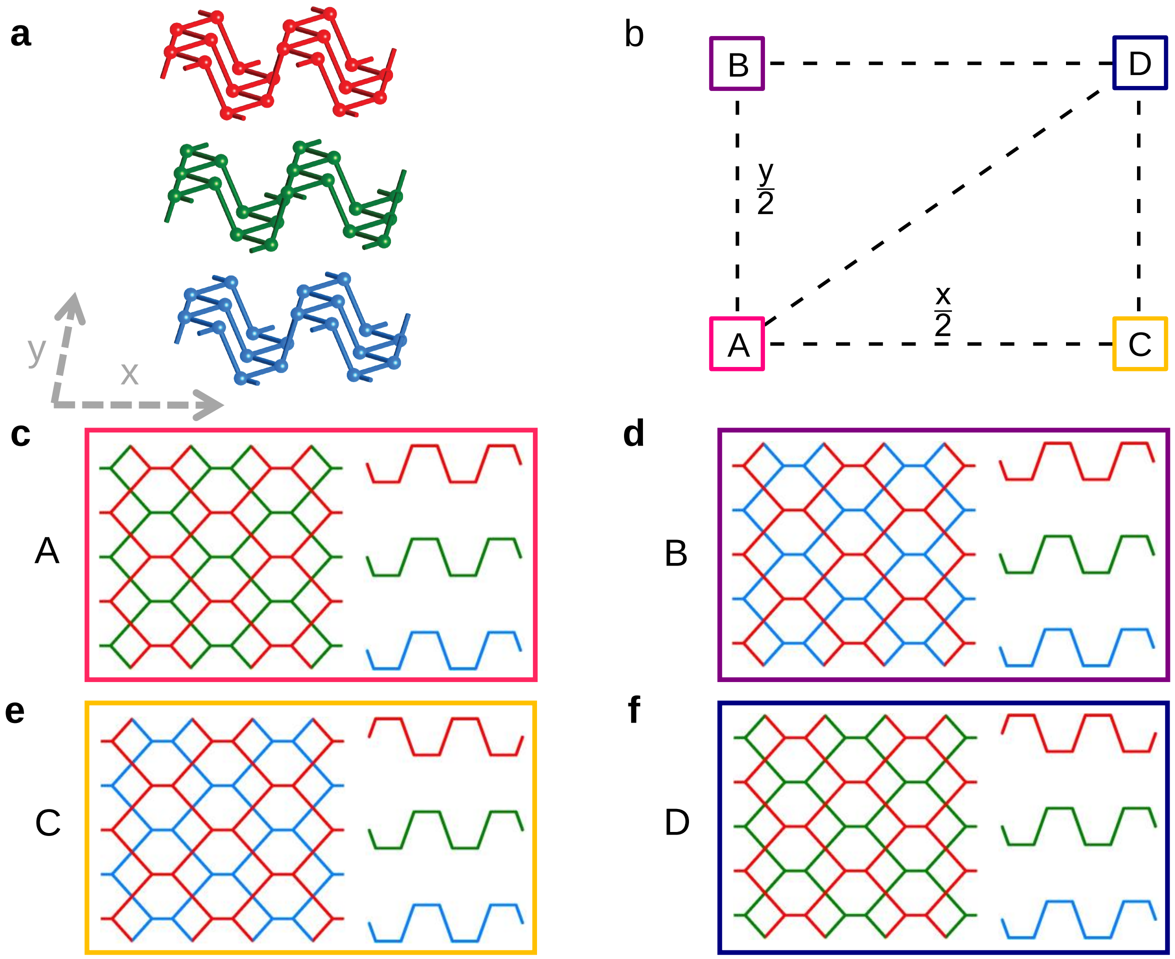


Supplementary Fig. 3. **Schematic illustration of the high-symmetry stacking orders for monolayer/bilayer phosphorene heterostructures**. Generally, the ABX (X for A, B, C, or D) stacking order labels the relative position of the top monolayer (X layer) compared to the bottom A or middle B layer. **a**, The schematic representation of ABA stacking. The blue, green and red indicate the bottom, middle, and top layers in ABA stacking, respectively. In addition, the armchair and zigzag direction are labeled as x- and y-direction, respectively. **b**, Four high-symmetry stacking orders in monolayer/bilayer phosphorene structures. Generally, intrinsic trilayer phosphorene takes ABA staking (pink), as shown in **a**. When the top monolayer is translated by ½*y* along the y-direction, the result configuration is labeled as ABB stacking (purple). Similarly, ABC stacking (orange) forms if the translation is performed along x-direction with the length of

½*x*. In addition, the ABD stacking (dark blue) forms when the top monolayer is shifted along the diagonal direction of phosphorene lattice. The corresponding atom configurations of ABA, ABB, ABC, and ABD stacking orders are shown in **c**, **d**, **e**, and **f**, respectively. The red, green and blue colors in **c**–**f** indicate the top, middle, and bottom layer of phosphorene, respectively. Moreover, the left and right panel in **c**–**f** represent the top-view and side-view of these structures.

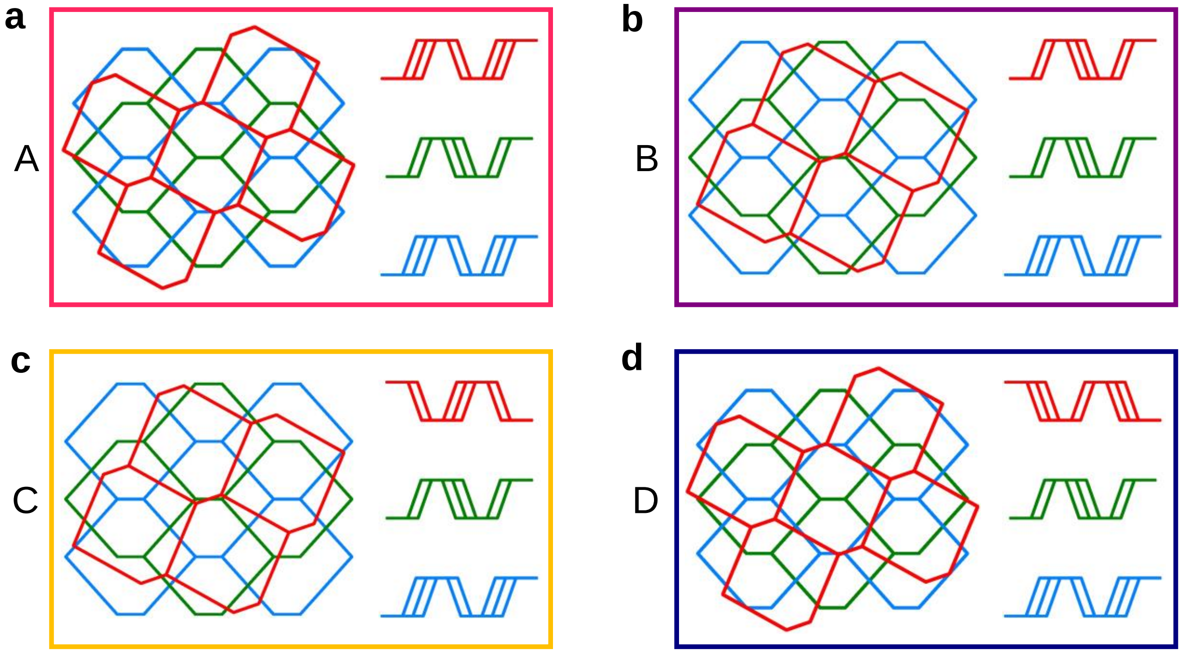


Supplementary Fig. 4. **Schematic illustration of the atom configurations for four special stacking configurations in the 20° twisted monolayer/bilayer phosphorene heterostructure.** **a**–**d**, The atom configurations (left, top-view, right, side-view) of the near-ABA (**a**), near-ABB (**b**), near-ABC (**c**), and near-ABD (**d**) stacking that are labeled as A, B, C, and D configurations in the main text, respectively. The red, green, and blue color in **a**–**d** represent the top, middle, and bottom layer of the 20° twisted phosphorene heterostructure, respectively.

Supplementary Note 3. Identification of twisted monolayer/bilayer phosphorene heterostructures

Supplementary Fig. 5a, 5b, and 5c show the optical images of monolayer, bilayer phosphorene (before stacking), and their twisted heterostructure, respectively. The red and blue dashed lines represent the monolayer and bilayer phosphorene, respectively. The monolayer phosphorene broke during transfer, giving rise to the irregular shape shown in Supplementary Fig. 5c and 5d. We further identified different regions by mapping their PL emissions at 77 K. As an example, Supplementary Fig. 5e shows the bilayer region in device D1, which matches well with the optical images shown in Supplementary Fig. 5d. Markedly, strong PL emission was observed from the twisted heterostructure region, as shown in Supplementary Fig. 5f. Moreover, the strongly quenched area of the bilayer phosphorene matched well with the PL emission area of the twisted structure, indicating the formation of a heterostructure.

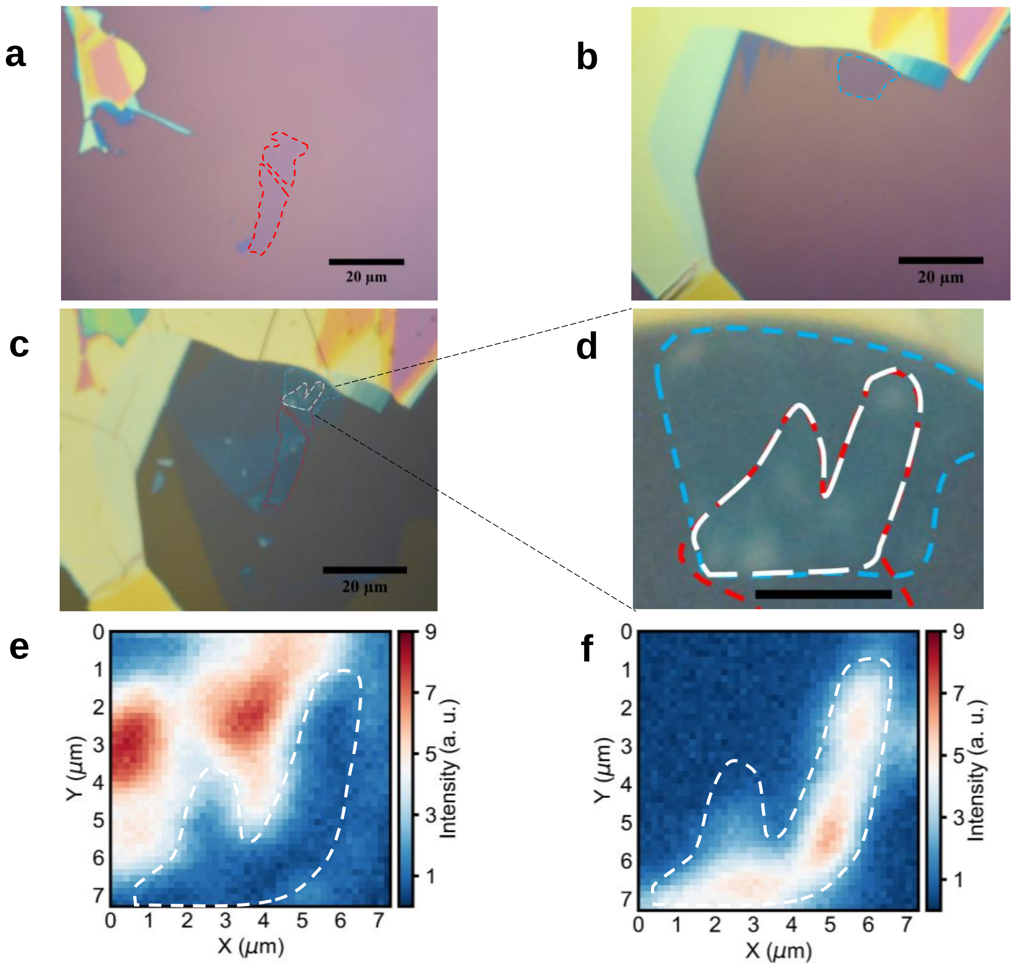


Supplementary Fig. 5. **Identification of twisted monolayer/bilayer phosphorene heterostructures.** The optical images of the monolayer (**a**), bilayer phosphorene (**b**) before stacking, and their twisted structure (**c**). Red and blue dashed lines indicate the monolayer (**a**) and bilayer phosphorenes (**b**), respectively. The monolayer phosphorene broke during transfer, giving rise to the irregular shape. **d**, The zoom-in image of **c** with the twisted phosphorene heterostructure highlighted by the white dashed line. **e** and **f**, The PL mapping results using PL emission peaks of bilayer (**e**) and twisted phosphorene heterostructure (**f**). The PL quenching region in **e** matches well with the emission region of the twisted phosphorene heterostructures, as shown in white dashed lines.

Supplementary Note 4. Angle determination for twisted monolayer/bilayer phosphorene heterostructures

Two more devices (D2 and D3) were fabricated using the same method described in the main text. Supplementary Fig. 6a and 6b show the optical image of D2 and D3, respectively. The white dashed and solid lines indicated the monolayer and bilayer phosphorene regions, respectively.

We fit the experimental angle-dependent PL emission spectra with the function:

$$I = I_0 * cos^2(\theta + \varphi) + C \quad (1),$$

where $I$ represents the PL emission intensity, $I_0$ and $C$ are constants to be fitted, $\theta$ and $\varphi$ indicate the incident angle of polarized light and the phase angle, respectively. Based on this method, the relative angle difference between monolayer and bilayer in device D1, D2 and D3 are 19° ± 1.1°, 5.9° ± 0.8°, and 2.2° ± 0.9°, respectively, see Fig. 1e in the main text, Supplementary Fig. 6c and 6d.

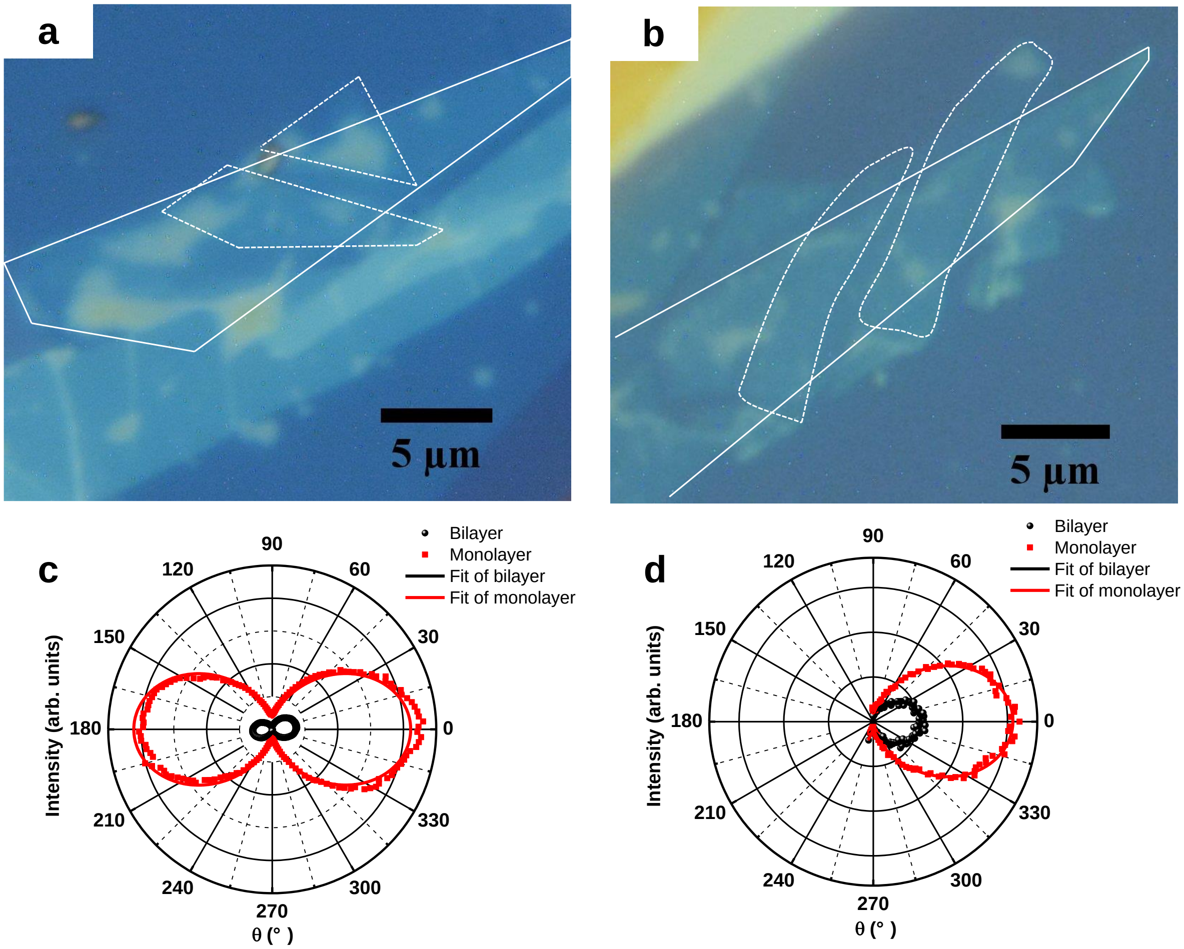


Supplementary Fig. 6. **Characterization of D2 and D3**. **a**,**b**, the optical images of D2 (**a**) and D3 (**b**). The white dashed and solid lines indicate the monolayer and bilayer phosphorene, respectively. **c**,**d**, angle-dependent PL emission of the monolayer (red square) and bilayer phosphorene (black sphere) in D2 (**a**) and D3 (**b**). The red and black solid lines represent the fitting curves of angle-dependent PL emission for monolayer and bilayer phosphorene, respectively.

Supplementary Note 5. Relationship between the twist angle and lowest energy transitions

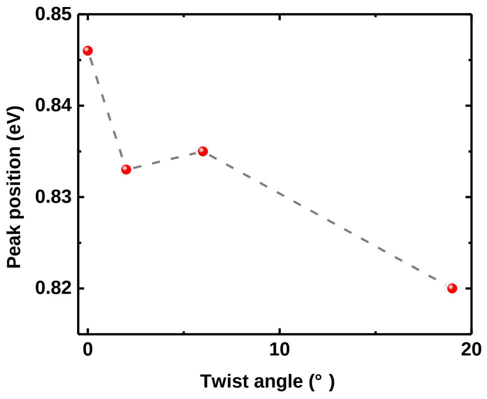


Supplementary Fig. 7. **The experimental peak positions of twisted monolayer/bilayer phosphorene heterostructures with different twist angles.**

The experimental results indicate that monolayer/bilayer phosphorene heterostructures with different twist angles show emerging optical transitions at ~0.83 eV with small energy variations.

Supplementary Note 6. Determine the primary optical axis for the 20° twisted monolayer/bilayer phosphorene heterostructure.

A 20.04° (referred as 20° for simplicity) commensurate twisted phosphorene heterostructure was used to model our experimental 19° one. The super cell of the 20° twisted monolayer/bilayer phosphorene heterostructure was constructed by two superlattice vectors $\mathbf{x} = 4\mathbf{a}_1+\mathbf{b}_1$ and $\mathbf{y} = -\mathbf{a}_1+8\mathbf{b}_1$, where $\mathbf{a}_1$ and $\mathbf{b}_1$ are the primitive lattice vectors of the monolayer phosphorene in the real space (Supplementary Fig. 8). Therefore, the x-direction (labeled as x) of the 20° twisted phosphorene heterostructure laid in the middle of the armchair direction of its constituent monolayer (-10°) and that of bilayer phosphorene (10°), as shown in Supplementary Fig. 8.

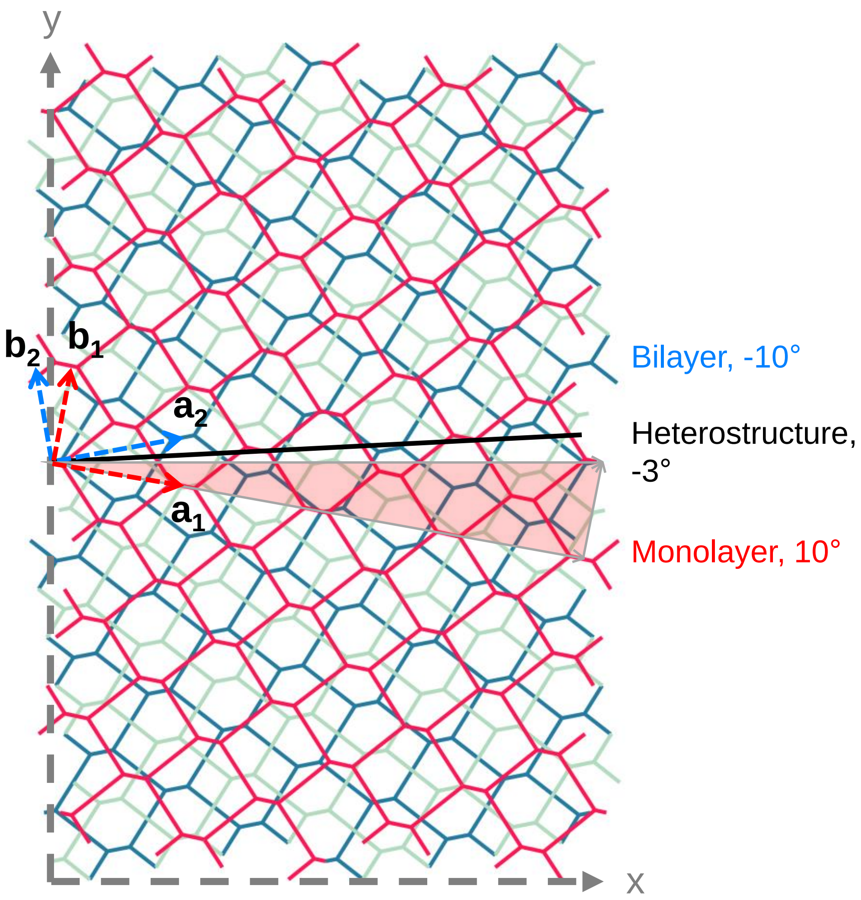


Supplementary Fig. 8. **The supercell of the 20° twisted heterostructures**. The armchair directions of constituent monolayer (red dashed arrow, $\mathbf{a}_1$) and bilayer phosphorenes (blue dashed arrow, $\mathbf{a}_2$) are at 10° and -10° with respect to x-axis (x) of the supercell, as determined by their dielectric function matrix. The red triangle demonstrates the construction of the superlattice vector **x** for the heterostructure using the lattice vector of monolayer phosphorene, as labeled as $\mathbf{a}_1$ and $\mathbf{b}_1$. The calculated optical principal axis of the heterostructures (black solid line) lies at -3° with respect to the x-axis of the supercell.

The dielectric function matrix of the heterostructure can be expressed as $M = \begin{pmatrix} \varepsilon_x & \varepsilon_{xy} \\ \varepsilon_{xy} & \varepsilon_y \end{pmatrix}$, and its two eigenvectors indicate the polarization directions for the heterostructure and the eigenvalues

represent the dielectric function along the corresponding directions. The calculated dielectric functions for 20° twisted heterostructure had non-zero components along xy-direction (blue lines, Supplementary Fig. 9), which indicated the primary optical axis of the twisted heterostructure was away from its x-direction. Solving the dielectric matrix at the energy of 2.33 eV, it gives rise to the polarization direction at -3.1° with respect to the x-axis of the Cartesian coordinate system, where the armchair (polarization) directions of the constituent bilayer and monolayer phosphorene lie at -10° and 10°, respectively (see Supplementary Fig. 8). We then conclude that the polarization axis of the heterostructure is closer to that of the bilayer with an angle difference of 7°.

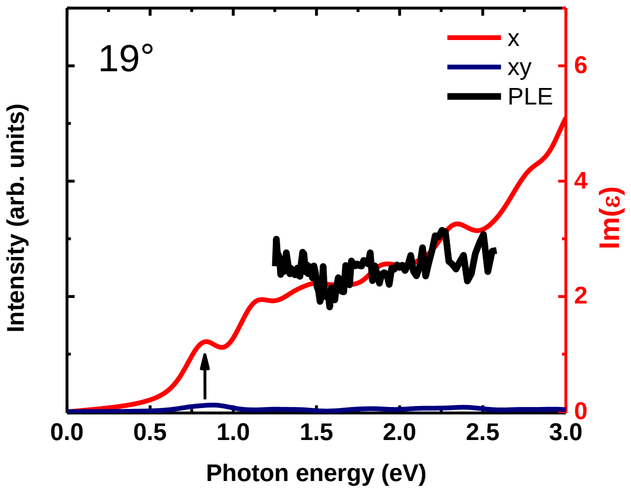


Supplementary Fig. 9. **The x- (red) and xy- (blue) component of the calculated dielectric function of the 20° twisted monolayer/bilayer phosphorene heterostructure.** The non-zero xy-component can be easily identified.

Supplementary Note 7. Calculated band structures and the imaginary part of dielectric functions for few-layer phosphorenes.

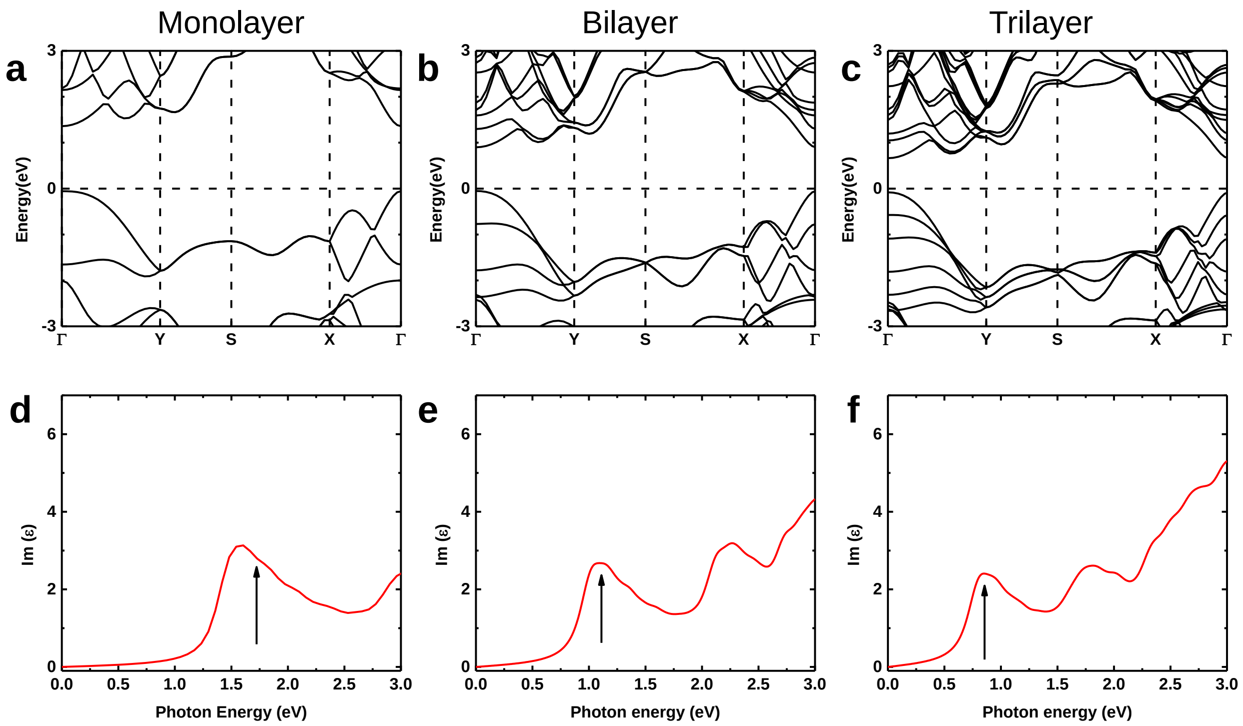


Supplementary Fig. 10. **Calculation results of intrinsic few-layer phosphorenes.** The calculated band structures (**a**–**c**) and the imaginary part of dielectric functions (**d**–**f**) for monolayer (**a**,**d**), bilayer (**b,e**), and trilayer phosphorene (**c**,**f**) using mBJ functional. The black arrow in **d**–**f** indicates the PL emission energy of monolayer (**d**), bilayer (**e**) and trilayer phosphorene (**f**). In addition, the above bandgap transitions can be identified for both bilayer (**e**) and trilayer phosphorene (**f**)[1,2], respectively.

Supplementary Note 8. Partial charge density distributions of few-layer phosphorenes

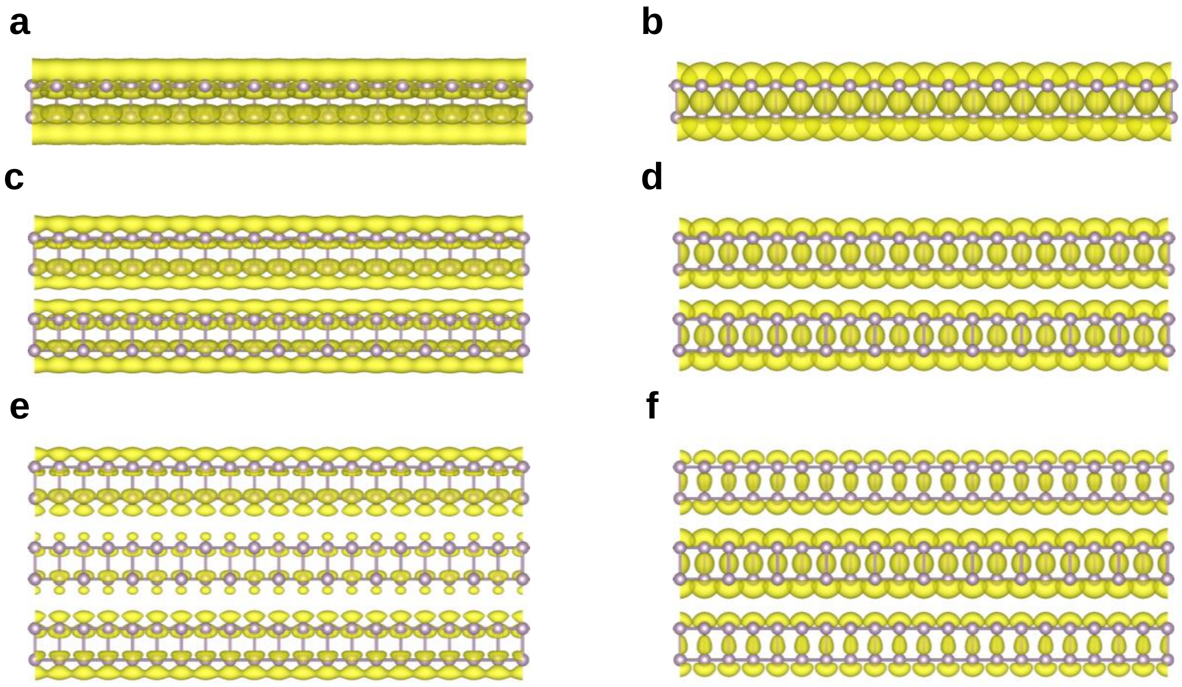


Supplementary Fig. 11. **Calculated Partial charge density distribution of few-layer phosphorenes.** The partial charge density distribution for the CBM (**a**,**c**) and VBM (**b**,**d**) of monolayer (**a**,**b**), bilayer (**c**,**d**), and trilayer phosphorenes (**e**,**f**), respectively. All the isosurface levels are set to be $3.5 \times 10^{-3}$ e$Å^{-3}$.

Supplementary Note 9. Calculated properties of twisted monolayer/bilayer phosphorene heterostructures

The interlayer coupling strength varies with different stacking configurations, which gives rise to different optical properties. Supplementary Fig. 12a shows the calculated optical properties of trilayer phosphorenes with four high-symmetry configurations, namely ABA, ABB, ABC, and ABD stackings (without rotation, Supplementary Fig. 3). The ABA and ABB stacking

configurations show similar optical transition features, with transition energies and coupling strengths slightly different. Likewise, the optical transitions in ABC and ABD stackings are also similar. However, there are profound differences between the optical transitions in ABA/ABB and those in ABC/ABD stacking configurations. The lowest energy transition peaks of ABA and ABB stacked trilayer phosphorene locate at ~0.8 eV, whereas those of ABC and ABD stacked trilayer phosphorene lie at ~0.5 eV. We further show the electronic bandgap and band alignment of the ABA, ABB, ABC, and ABD stacking regions in Supplementary Fig. 12b and Supplementary Fig. 12c, respectively.

In small twist-angle moiré heterostructure, optical transitions with different stackings will contribute. It seems that the ABA and ABB transitions at ~0.8 eV will dominate the overall absorption peaks because they have a larger optical transition. For photoluminescence, there should be a lower energy emission close to optical transitions at ~0.5 eV in ABC and ABD region, but that is outside of our experimental measurement range. For larger twist angles, the coupling between regions of different stackings becomes important and the optical spectra cannot be easily attributed to optical resonances from individual stacking orders.

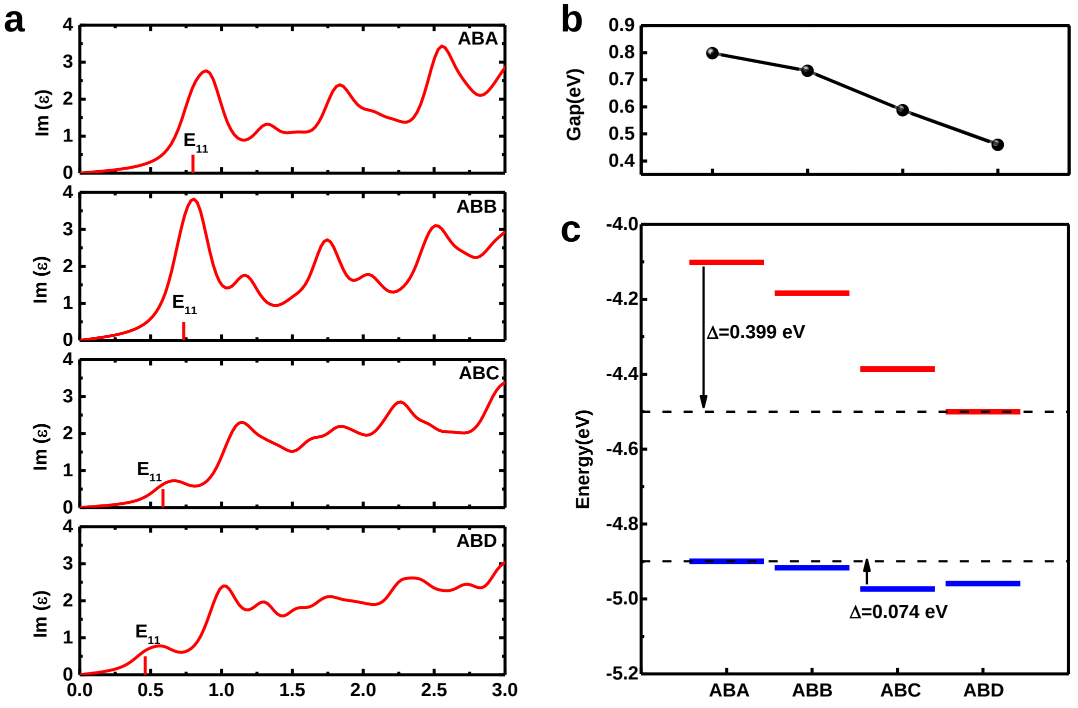


Supplementary Fig. 12. **The calculated optical properties of four stacking configurations, namely ABA, ABB, ABC, and ABD stacking.** The calculated imaginary part of dielectric function (**a**), band gap (**b**), and band alignment (**c**) of four stacking configurations. Here, all calculations were performed using accurate hybrid HSE functional.

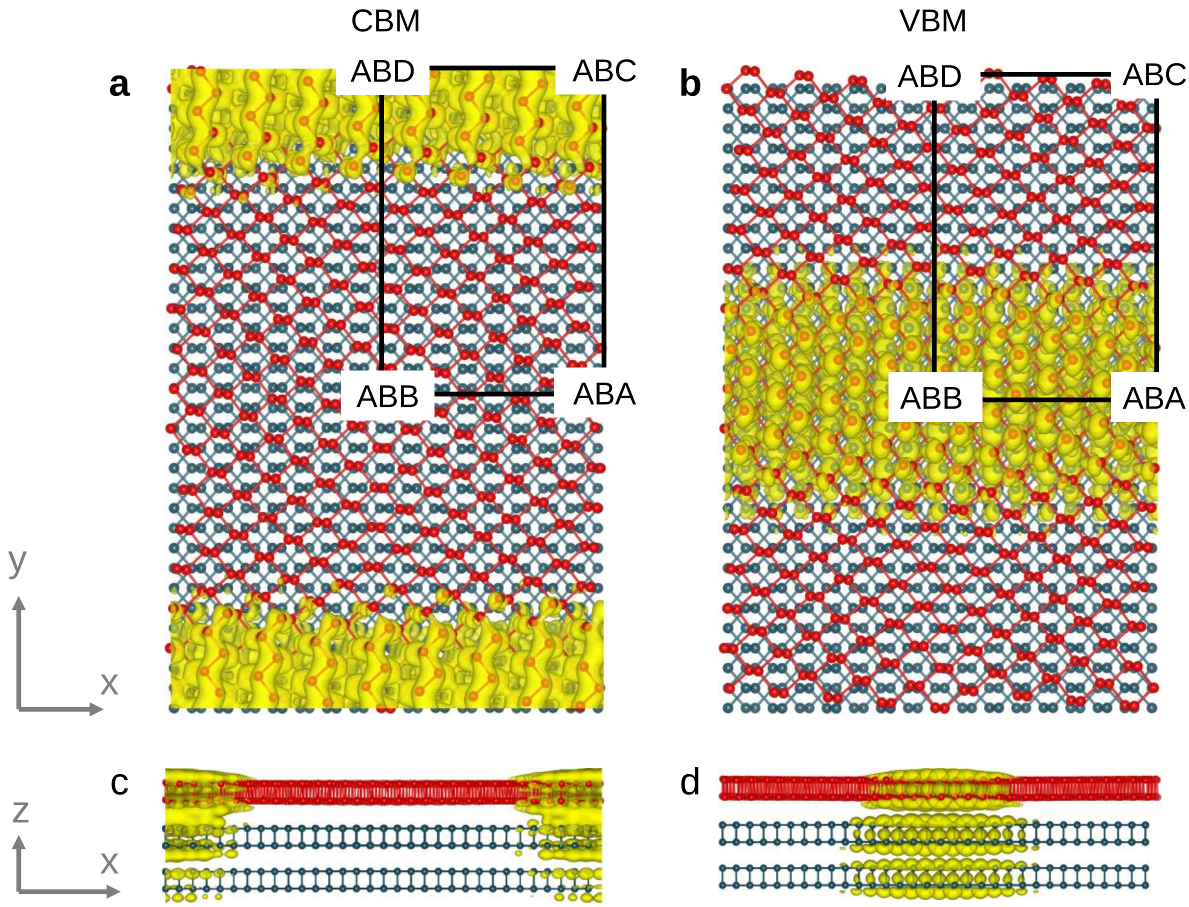


Supplementary Fig. 13. **The calculated partial charge density distributions of a 5° twisted monolayer/bilayer phosphorene heterostructure**. The top (**a** and **b**) and side (**c** and **d**) views of the partial charge density distributions for the CBM (**a** and **c**) and VBM (**b** and **d**) of the twisted monolayer/bilayer phosphorene heterostructure. The isosurface levels are set to be $3\times10^{-11}$ eÅ$^{-3}$. Here we use RESCU software[3] with real-space KS-DFT method to calculate the charge density of 5° twisted monolayer/bilayer phosphorene heterostructure with 1540 atoms.

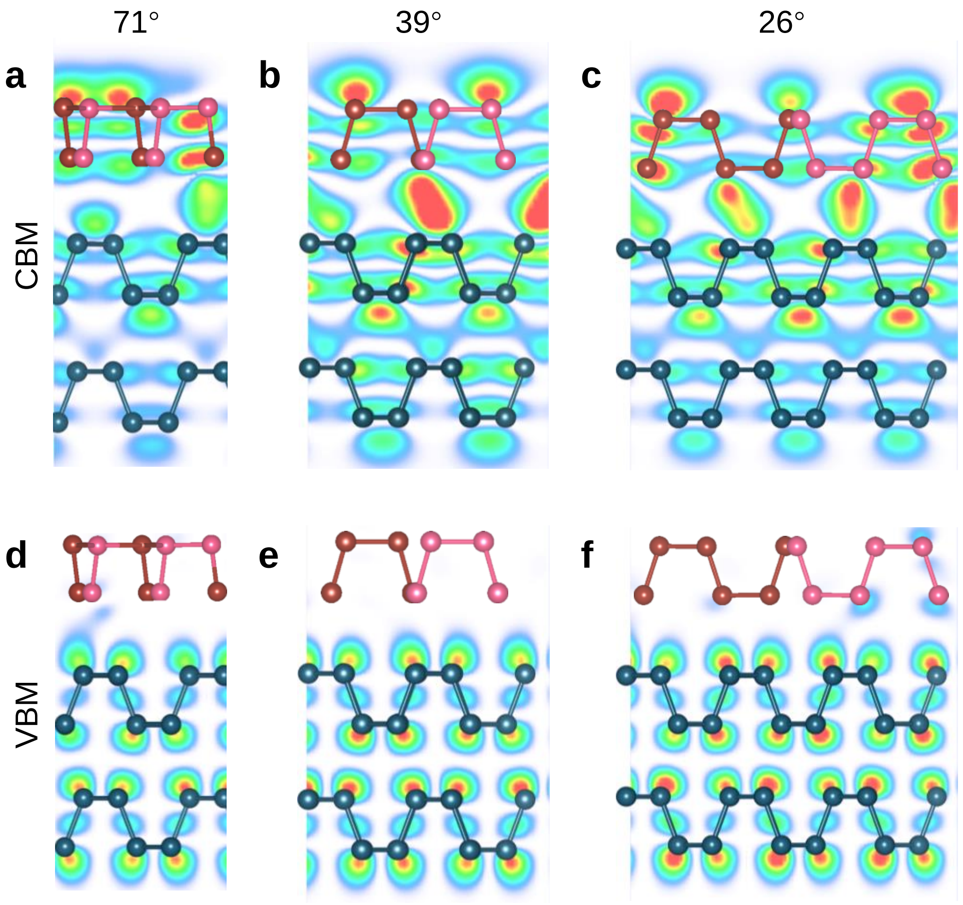


Supplementary Fig. 14. **The sectional partial charge density distributions at the CBM (top panels) and VBM (bottom panels) of twisted monolayer/bilayer phosphorenes with the angle of 71° (a and d), 39° (b and e), and 26° (c and f).** Brown and pink indicate phosphorus atoms in front of and behind the sectional plane, respectively. The isosurface levels are set to be $1.5\times10^{-9}$ $eÅ^{-3}$. The wavefunctions of CBM mainly distribute at the interfaces between monolayer and bilayer phosphorene, whereas, they are localized in the bottom bilayer for VBM.

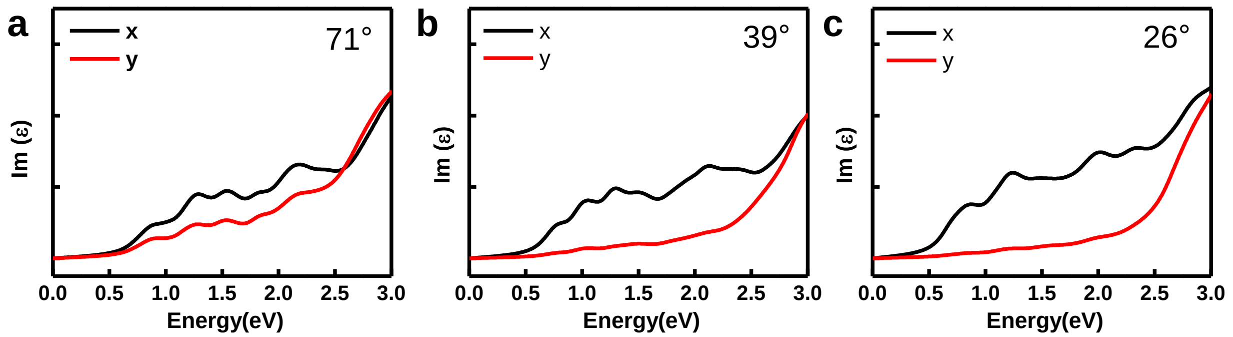


Supplementary Fig. 15. **The calculated imaginary parts of dielectric functions for the 71° (a), 39° (b), and 26° twisted phosphorene heterostructures (c).** The black and red lines indicate the optical absorption along x and y direction of the superlattices, respectively.

## Supplementary References

1 Li, L. *et al.* Direct observation of the layer-dependent electronic structure in phosphorene. *Nat. Nanotechnol.* **12**, 21-25 (2016).

2 Qiao, J., Kong, X., Hu, Z.-X., Yang, F. & Ji, W. High-mobility transport anisotropy and linear dichroism in few-layer black phosphorus. *Nat. Commun.* **5**, 4475 (2014).

3 Michaud-Rioux, V., Zhang, L. & Guo, H. RESCU: A real space electronic structure method. *J. Comput. Phys.* **307**, 593-613 (2016).
